# Fully closed cell sorter for immune cell therapy manufacturing

**DOI:** 10.1016/j.omtm.2023.07.012

**Published:** 2023-08-02

**Authors:** Masahiro Matsumoto, Shinji Tashiro, Tatsumi Ito, Kazuya Takahashi, Gakuji Hashimoto, Junji Kajihara, Yoshihiro Miyahara, Hiroshi Shiku, Yoichi Katsumoto

**Affiliations:** 1 Tokyo Laboratory 11, R&D Center, Sony Group Corporation, Tokyo, Japan; 2 Department of Personalized Cancer Immunotherapy, Mie University Graduate School of Medicine, Mie, Japan; 3 Center for Comprehensive Cancer Immunotherapy, Mie University, Mie, Japan; 4 Department of Cellular and Molecular Immunology, Mie University Graduate School of Medicine, Mie, Japan

**Keywords:** cell therapy manufacturing, cell sorting, cell purification, functional assay, cGMP, cell therapy, cell sorter, flow cytometry

## Abstract

By analyzing patients treated with adoptive immune cell therapies, various immune cell phenotypes have been found in the starting and infused materials as determinants of sustained remission. The isolation of these specific phenotypes for clinical use requires current Good Manufacturing Practice (cGMP)-compliant cell-sorting technologies with multiparameter selection capabilities. Here, we developed a cGMP-requirement-applicable fully closed cell sorter that has a suction mechanism and multiparameter detection using two laser optical settings. Negative pressure generated by a change in the chamber volume at a sorting point allows the isolation of cells of interest with high viability and purity. Our study demonstrated that this microfluidic sorter enables the isolation of cells of interest at an effective rate of 7,000 sorts per second on average. A purity of 85.5% and 77.1% effective yield with 93.7% viability was obtained when applying a target population of 35.9% in total (lymphocyte+CD8+) at 15,000 events per second (2 × 10^7^ cells/mL). The sorted gene-modified T cells maintain largely unaltered proliferation, antigen recognition, cytokine release, and cytotoxicity functionalities.

## Graphical abstract

**Figure:**
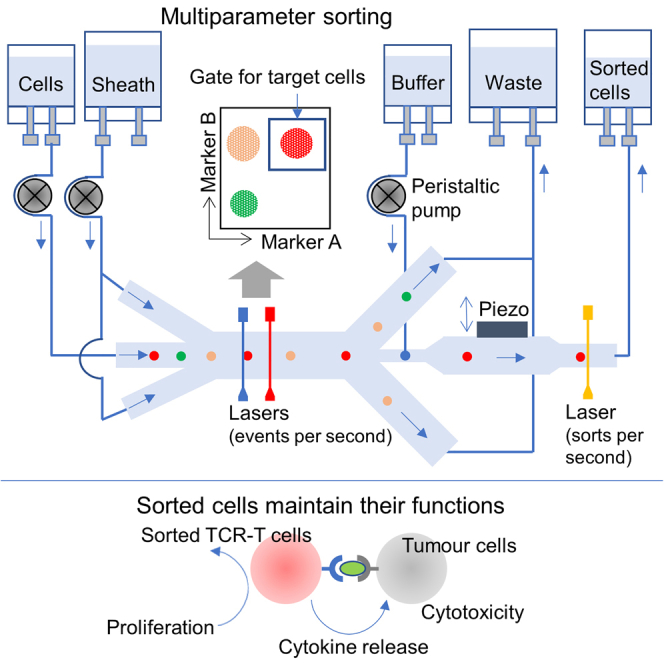

## Teaser

Masahiro and colleagues developed a fully closed microfluidic chip cell sorter with a suction mechanism that can meet cGMP regulations and demonstrated its sorting performance. This sorter enables the isolation of cells using multiple parameters to provide high purity and high effective yield. The sorted cells maintained their functionality.

## Introduction

There is growing interest in the use of immune cell therapy to treat many diseases such as tumors (hematologic and solid), autoimmune diseases, and infectious diseases.^1^^,^^2^ Various chimeric antigen receptors (CARs) or T cell receptors (TCRs) expressing immune cells such as T cells, natural killer (NK) cells,^3^ NKT cells,^4^ regulatory T cells,^5^ and macrophages^6^ are currently being developed and tested in clinical studies. However, many challenges remain with respect to improving efficacy, reducing side effects, and treating solid tumors.^7^^,^^8^ Although structures of gene-modified cells such as an antigen-binding domain, a hinge, a transmembrane domain, and an intracellular domain are important for improving the efficacy of such treatments and to sustain remission,^7^^,^^9^^,^^10^^,^^11^^,^^12^^,^^13^^,^^14^ cell phenotype is also important. Characterization of the cell types in the starting materials and in the infusions given to patients exhibiting sustained remission have shown a higher proportion of stem cell-like memory T (T_SCM_) and central memory T (T_CM_) cells,^15^ an elevated frequency of CD27+CD45RO-CD8+ T cells,^16^ and an increased frequency of CAR+ T helper type 2+ T cells.^17^ A defined ratio of CAR+CD4+:CAR+CD8+ (1:1) in patients with acute lymphoblastic leukemia and non-Hodgkin’s lymphoma showed synergistic enhancement in antitumor activity.^15^^,^^18^ Selective depletion of alloreactive cells following haploidentical transplantation and purification of gene-modified cells with suicide genes such as inducible caspase 9 are also important to reduce the risk of graft-versus-host disease (GvHD).^19^

To make full use of these results requires multiparametric cell isolation technologies that meet current Good Manufacturing Practice (cGMP) regulations. Magnetic-activated cell sorting (MACS) systems such as CliniMACS are used in cell therapy manufacturing.^20^^,^^21^ Although this system allows the isolation of up to order of approximately 10^8^ cells, several rounds of isolation are necessary if the cells of interest are identified by more than two markers. Such cases are addressed by negative selection using several markers followed by positive selection with one marker,^22^ as well as serial positive selection using Fab streptamers.^23^ However, it is time consuming to incubate the cells at each step with antibodies or Fabs. Fluorescence-activated cell sorting (FACS) is widely used to achieve multiparameter selection. However, a traditional FACS system generates droplets in air (droplet cell sorter), but this open system is not suitable for isolating cells for clinical use due to potential contamination of the environment, the system, and/or the sample. This drawback has been addressed using cell isolation technologies based on microfluidic chips inside which the cells are sorted.^24^^,^^25^ Fluidic switching technology is used to change the flow direction of the cells of interest into a collection channel. This approach does not use a sheath fluid to protect the cells from mechanical stress at high flow rate, and thus parallelization of the flow streams is necessary to achieve a high-throughput cell-sorting rate.^25^ However, there is a variation in the fluid velocity and excitation intensity in each channel, and fluorescence signal crosstalk needs to be taken in to account. To date, no microfluidic cell sorter with the same optical setting and performance as traditional FACS has achieved an adequate sorting rate and high cell purity and viability. Sorting performance, and especially sorting rate, has been tested using a low target population (less than 5% of the population), and there are no reports of the recovery or effective yield or of the functional analysis of the sorted cells.

Here, we report a cGMP-requirement-applicable cell-sorting technology using a microfluidic chip that enables the isolation of cells using multiple parameters to provide high purity and high effective yields of cells at an adequate acquisition rate. The system utilizes two excitation lasers and has the same sensitivity as traditional FACS. Here, we isolated NY-ESO-1 TCR-modified T cells and confirmed that the sorted cells maintained their functionality, such as proliferation, antigen recognition, cytokine release, cytotoxicity, and viability, better than that following sorting using traditional FACS.

## Results

### Fully closed microfluidic chip cell sorter and its sorting mechanism

Our fully closed microfluidic chip sorting system has peristaltic tube pumps and is compatible with the sterile connection of bags that supply the sample, sheath, and gate fluid using a tube connection device, enabling us to meet cGMP regulations. All fluid parts including microfluidic chip, tubes, and bags are single-use disposable, eliminating the risk of sample contamination and carryover. The inline filter between the sample inlet and the microfluidic chip can prevent the occurrence of clogging in our system. The scatter-based clog detection and recovery technology is implemented. With this, the clogs are automatically detected and disintegrated by controlling the fluidics, ensuring robust operation over long sorting times. These features relating to our technology are summaried in Table 1.

**Table 1:** The summary of our technology in comparison to droplet sorter

	Droplet sorter (traditional cell sorter)	Microfluidic chip sorter (our system)
Optics	laser excitation, forward- and side-scatter detection, fluorescence detection (up to 13–18 markers) ∗BD FACSAria I ∼ III	laser excitations, forward- and back-scatter detection, fluorescence detection (up to 8 markers)
Fluidics	hydrodynamic focusing for detection	hydrodynamic focusing for detection
	open system to generate droplets in air (jet-in-air)	closed system to acquire cells of interest in microfluidic chip
	cleaning is required for fluidic parts	all fluidic parts are single-use disposable
	inline filter is not normally implemented	inline filter is implemented
Others	–	clog detection and recovery technology

**Figure 1:**
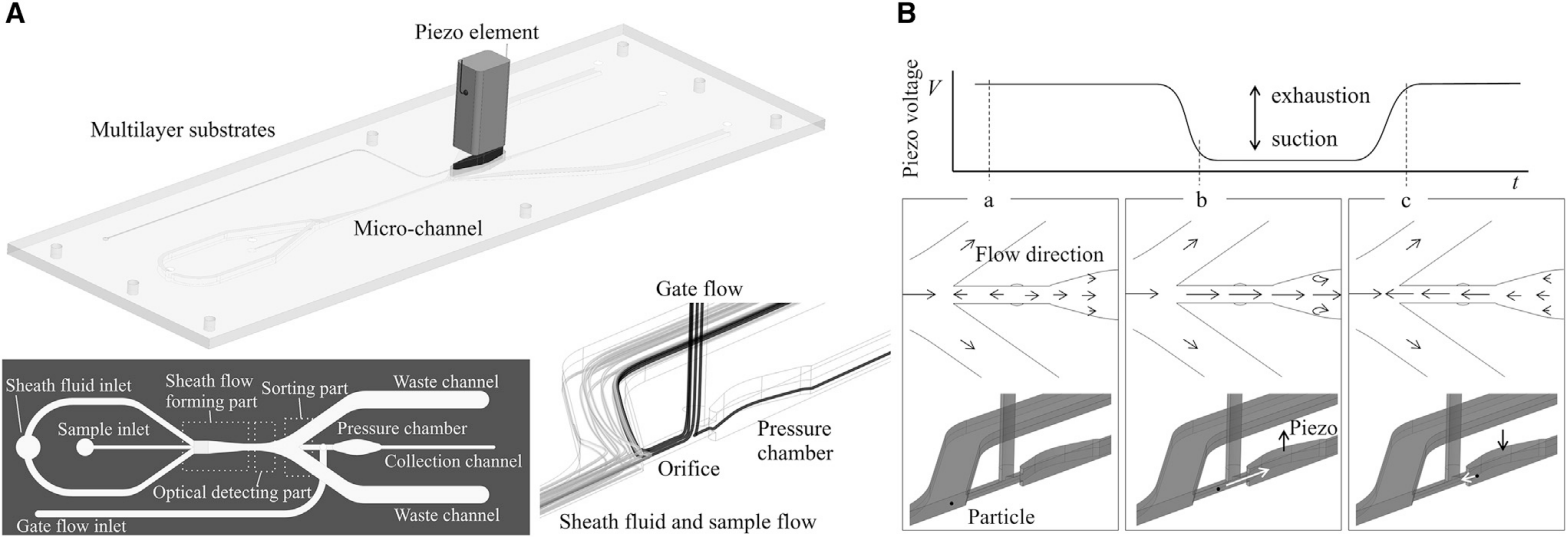
Schematic illustration of the microfluidic chip sorter and its sorting mechanism (A, top) Microfluidic chip cell sorter with a piezo element on a pressure chamber. (A, bottom left) Schematic design of the chip comprising a sheath flow part, optical detection part, and sorting part. (A, bottom right) Flow streamlines of sheath flow, sample flow, and gate flow. (B, top) A representative waveform applied to the piezo element to acquire a target particle. (B, bottom left) The upstream flow generated by gate flow prevents particles from entering the collection chamber. (B, bottom middle) Pressure chamber volume changes and negative pressure is generated to acquire particles of interest when a driving voltage is applied. When the applied voltage is released, the piezo element returns to the original position. Upstream flow is generated at that time (B, bottom right), and then the flow state returns quickly to the original state (B, bottom left).

The microfluidic chip was fabricated by multilayer bonding of COP (cyclo-olefin polymer; Zeonex480, Zeon Corporation) mass-produced substrate which has microchannels, with a piezo element on the pressure chamber to actuate the flow. The chip has three main functional parts: sheath flow formation, optical detection, and sorting (Figure 1A). First, the cells are injected into a sheath flow stream, where they are forced to accelerate and flow into the center of the stream, allowing them to align in a single file in the direction of flow. Two lasers (488 and 637 nm) are used as excitation sources to detect forward-scattered (FSC) signals, the back-scattered (BSC) signals, and the fluorescence signals by photomultipliers. This optical setting allows measurement of the detection rate and the flow rate of each particle and adjustment of the piezo operation start time (delay time), an important parameter for sorting the cells of interest. Finally, the flow containing the cells reaches the central collection channel flanked by waste channels, where the flow forms a stagnation point. A separate flow (gate flow) in both the upstream and downstream directions is generated at the channel connecting to the pressure chamber. The upstream gate flow prevents the cells from entering the pressure chamber when a piezo element is not actuated (Figure 1B, bottom left). As a cell of interest enters the sorting part of the device, at a certain time after this cell is detected at the optical detection part, a driving voltage is applied to the piezo element to change the volume of the pressure chamber and to generate a negative pressure, allowing the cell to enter the pressure chamber and reach the collection channel (Figure 1B, bottom middle). Backward flow is generated when the applied voltage is released and the piezo element returns to its original position (Figure 1B, bottom right). Then, the flow state returns quickly to the original state, enabling the cell to flow into the collection chamber by the downstream flow generated by the gate flow (Figure 1B, bottom left). A laser is used as an excitation source to detect FSC signals of sorted cells, enabling us to count the number of cells sorted. Optimal cell sort setting can be automatically adjusted in our system by counting the number of cells sorted. Optimal cell sorting requires precise design and control of the volume of the pressure chamber, the flow rate of the sheath and gate flows, and the waveform and voltage of the piezo element.

### Sorting performance

**Figure 2:**
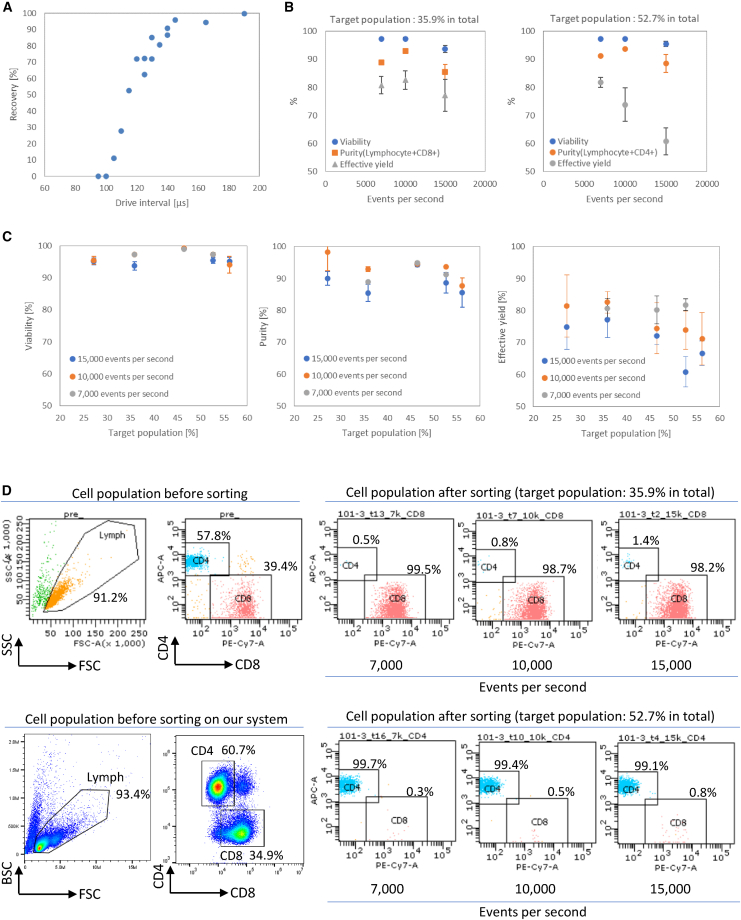
Sorting performance of the microfluidic chip sorter (A) The recovery at various interval times “t” was confirmed by applying a fake acquisition operation prior to the true operation, using 5 μm polystyrene particles. (B) The viability, purity, and effective yield at various event rates with two different target populations using PBMCs. Solid circles show viability, solid rectangles show purity, and solid triangles show effective yield. n = 3, 3, and 13 at 7,000, 10,000, and 15,000 events per second, respectively, at the target population of 35.9% (sort gate: lymphocyte+CD8+). n = 3, 3, and 7 at 7,000, 10,000, and 15,000 events per second, respectively, at the target population of 52.7% (sort gate: lymphocyte+CD4+). Error bars represent standard deviation of the mean. (C) The viability, purity, and effective yield at various target populations with three different event rates using PBMCs (n > 3). Error bars represent standard deviation of the mean. (D) Representative flow cytometry data before and after sorting at three different event rates, used two different target populations.

The operation of drawing the fluid mass containing the target cells at the stagnation point into the pressure chamber is necessary to acquire cells of interest, and the upper limit of the particle acquisition speed is defined by the time required for the fluid field disturbed by the operation to settle down. The particle acquisition speed was evaluated using 5 μm polystyrene particles by applying a fake acquisition operation prior to the true operation, allowing us to measure for how long the fake operation affects the true operation. This approach was necessary because it is difficult to control the distance between particles in single file precisely due to the randomness based on the Poisson distribution. Here, if a target was detected, the acquisition operation was carried out twice. The first acquisition was fake prior to the target reaching the sorting part of the device with an interval of duration “t.” The second acquisition was true acquisition to obtain the target. Recovery was calculated by dividing the number of particles acquired by the number of times the second acquisition was operated. The recovery increased rapidly at interval times of more than 100 μs (Figure 2A). A recovery of more than 90% was achieved at interval times longer than 140 μs, meaning that the acquisition rate was approximately 7,000 sorts per second on average.

Next, the viability, purity, and effective yield were evaluated using human peripheral blood mononuclear cells (PBMCs) at average event detection rates of 7,000, 10,000, and 15,000 events per second. The event detection rate was changed by increasing the sample flow rate from 21 to 45 μL/min, using the same concentration of 2 × 10^7^ cells/mL. One million cells were sorted during each experiment (n > 3). A total target population of 35.9% (lymphocyte+CD8+) provided 88.9% ± 0.05% purity and 80.7% ± 3.11% effective yield with 97.2% ± 0.19% viability at 7,000 events per second on average (Figure 2B, left). Effective yield was calculated by dividing the number of target cells sorted in the collection tube by the number of target cells acquired by the instrument. At 15,000 events per second, 85.5% ± 2.68% purity and 77.1% ± 5.62% effective yield with 93.7% ± 1.31% viability were obtained. A total target population of 52.7% (lymphocyte+CD4+) provided similar purity and viability at each event rate; however, the effective yield decreased as the event rate increased (Figure 2B, right). At 15,000 events per second, 88.6% ± 3.22% purity and 60.8% ± 4.73% effective yield with 95.5% ± 0.90% viability were obtained. The viability and purity were similar at each target population with three different events per second (Figure 2C, left and middle), and more than 94% viability and more than 85.5% purity were observed at each target population with different event rates. While the decrease in effective yield was not observed as the target population increased at 7,000 events per second, the effective yield decreased as the target population increased at 10,000 and 15,000 events per second (Figure 2C, right). Representative flow cytometry data before and after sorting at the three different event rates for two different target populations are shown in Figure 2D. Each population of lymphocyte+, CD4+, and CD8+ on our system is similar to that of a flow cytometry (Figure 2D, top left and bottom left).

### Functional analysis of sorted NY-ESO-1 TCR-modified T cells *in vitro*

**Figure 3:**
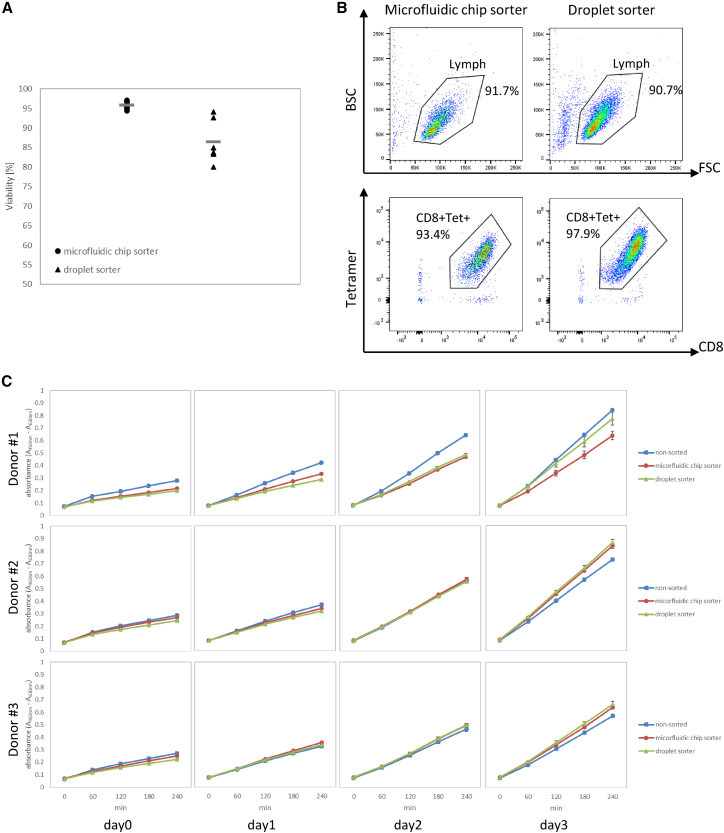
Viability and cell proliferation analysis using NY-ESO-1 TCR-T cells (A) The average viability is shown after sorting using the microfluidic sorter (n = 12) and droplet sorter (n = 6) at event rates from 1,000 to 1,300 events per second. Error bars represent standard deviation of the mean. (B) Representative flow cytometry data after sorting lymphocyte+CD8+Tet+ (tetramer+) TCR-T cells using the microfluidic sorter and droplet sorter. (C) The result of triplicate WST-1 assays on days 0, 1, 2, and 3 after sorting. Solid rectangles show the result for non-sorted (control) cells, solid circles show the results obtained using the microfluidic chip sorter, and solid triangles show the results obtained using the droplet sorter. Error bars represent standard deviation of the mean.

CD8+tetramer+TCR-T cells were sorted using our microfluidic chip sorter and an FACSAria I (BD Biosciences) droplet cell sorter device at event detection rates of between 1,000 and 1,300 events per second. The average viability of cells sorted using our microfluidic chip sorter was 95.8% ± 0.76%, which was higher than that of the droplet cell sorter (86.5% ± 5.12%) (Figure 3A). The purity of the sorted cells (lymphocyte+CD8+tetramer+) using our microfluidic chip sorter was 86.3% ± 0.57% (n = 3), similar to the 90.1% ± 1.00% purity (lymphocyte+CD8+tetramer+) achieved using the droplet cell sorter (n = 3). Representative flow cytometry data after sorting using our microfluidic sorter and droplet sorter are shown in Figure 3B.

The proliferation rates of the sorted cells were evaluated using the WST-1 assay reagent. The cell proliferation rate of both groups of sorted cells was similar to that of non-sorted cells, and the proliferation rate increased with increasing number of culture days after sorting (Figure 3C).

**Figure 4:**
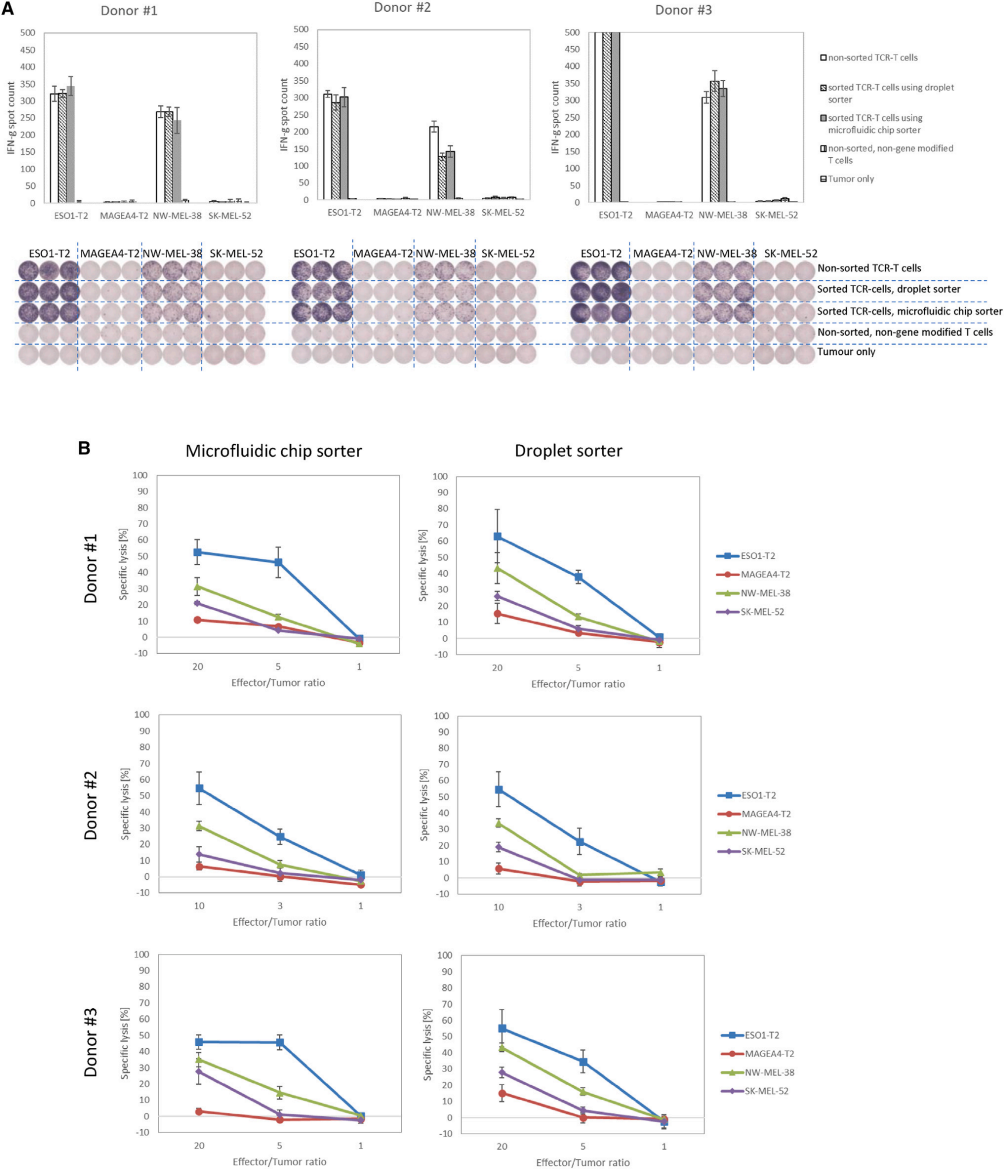
Cytokine secretion and cytotoxicity analysis using NY-ESO-1 TCR-T cells (A) IFN-γ spots were counted to show the potency of the cytokine produced by sorted TCR-T cells as determined by performing the ELISPOT assay in triplicate. Error bars represent standard deviation of the mean. The IFN-γ spot counts of all TCR-T cells from donor #3 treated with ESO1-T2 were saturated (A, right), and thus the number of counts was set to 500. (B) Cytotoxic activity of sorted TCR-T cells was shown by performing the ^51^Cr-release assay in triplicate. Error bars represent standard deviation of the mean. Solid rectangles show the results following reaction with ESO1-T2 (T2 cells pulsed with NY-ESO-1p157-165), solid circles show the results following reaction with MAGEA4-T2 (T2 cells pulsed with MAGE-A4p230-239 9), solid triangles show the results following reaction with NW-MEL-38, and solid diamonds show the results following reaction with SK-MEL-52.

Cytokine secretion potency was tested using the ELISPOT assay. The sorted cells recognized tumor cell lines (ESO1-T2 and NW-MEL-38) specifically, and interferon γ (IFN-γ) spots were observed (Figure 4A). A similar number of spots against ESO1-T2 and NW-MEL-38 was obtained in all wells treated with TCR-T cells, whereas no spots were observed in the other wells. The number of IFN-γ spots counted in donor #3 with ESO1-T2 was saturated and thus was counted as more than 500 spots. There was no difference between unsorted and sorted TCR-T cells.

Cytotoxic function was assessed using the chromium release assay. Specific lysis was observed against ESO1-T2 and NW-MEL-38 (Figure 4B), and the percentage of specific lysis of both groups of sorted TCR-T cells was similar.

## Discussion

In most immune cell therapies, the cell products infused into patients are unselected and thus the cell composition varies widely in both phenotype and subset.^26^^,^^27^ Recent studies have shown that the specific cell types and subsets of the starting materials and infusions are associated with long-term remission.^15^^,^^16^^,^^17^ Especially in solid tumors, regulatory T cell (Treg; CD25+CD4+) depletion using anti-CCR4 monoclonal antibody (mAb) or anti-CCR8 mAb is effective in reducing tumor-infiltrating effector Tregs (eTregs) and in enhancing antitumor activity,^28^^,^^29^ suggesting that depleting these cells from infusions could be advantageous. These results encouraged us to select specific cell types to improve efficacy and remission rate for treating both blood cancer and solid tumors. To select specific cell types as cell therapy infusions, multiparametric cell isolation technology would be effective, and we demonstrate our prototype of the cGMP-requirement-applicable microfluidic chip cell sorter. In this study, we evaluated our microfluidic chip cell sorter to confirm sorting performance and the functional properties of the sorted cells. While a piezo element with an operation rate of 87,000 actuations per second was implemented, the acquisition operation rate was approximately 7,000 sorts per second on average if 90% recovery was set as a threshold (Figure 2A). This indicated that the interval time, which is the time required for the fluid field to settle down after prior operation, is necessary to acquire cells of interest. The viability and purity were similar at each event rate and target population (Figures 2B and 2C). The effective yield decreased when a target population is around 50% at 15,000 events per second (Figure 2C, right). The 50% target population at 15,000 events per second means that target cells are flowing at 7,500 events per second on average (the interval time is less than 140 μs), which is higher than the highest acquisition rate of 7,000 sorts per second on average in the current setting, resulting in the decrease of the effective yield. This would explain the observed decrease in effective yield at 15,000 events per second using a 52.7% target population (Figure 2B, right) and highlights the importance of adjusting the event rate according to targeted percent population. The event rate can be adjusted by changing the sample flow rate on our system. In the case of 52.7% target population, the decrease of event rate from 15,000 to 10,000 events per second enabled us to increase the effective yield from 60.8% to 73.8% on average (Figure 2C, right). Although the higher cell concentration would be better to maximize the throughput, a cell concentration less than 2 × 10^7^ cells/mL is recommended to prevent cells from aggregating.

Approximately 4.4 × 10^7^ sorted cells were obtained from 2 × 10^8^ cells within 4 h (Figure 2B, left; 15,000 events per second, 35.9% target population, 93.7% viability, 85.5% purity, 77.1% effective yield, 2 × 10^7^ cells/mL, 45 μL/min sample flow rate). With a 52.7% target population, approximately 5.4 × 10^7^ sorted cells can be obtained within 4 h when starting with 2 × 10^8^ cells (Figure 2B, right; 15,000 events per second, 95.5% viability, 88.6% purity, 60.8% effective yield, 2 × 10^7^ cells/mL, 45 μL/min sample flow rate). Although the acquisition operation rate might not support obtaining >10^8^ sorted cells within several hours, we confirmed that the sorted cells maintained their ability to proliferate, release cytokines, and maintain cytotoxicity (Figures 3 and 4). This would enable us to isolate cells of interest in the middle of a cell culture or after gene transfer, followed by proliferation until the target number of cells is achieved. One advantage of this approach is that reagent costs of operating the cell selection device are cut, with an especially notable contribution from the cost of antibodies. Another advantage is that we can obtain cells with less antibody binding since, as the number of cells increases, antibodies dissociate during cell culture due to dilution into the culture medium. This may be particularly advantageous if the number of fluorophore-conjugated antibodies bound with infused cells needs to be reduced. In our system, sorted cells are collected in a gate flow with a flow rate of 0.2 mL/min. After 4 h of operation, the volume of the collection bag is 48 mL. Subsequent procedures such as centrifugation and buffer exchange are facile with this volume. Cell culture can proceed immediately after sorting if culture medium is used as the gate flow.

Sorting parameters can be adjusted automatically in our system, although the gate setting for the target population must be done manually. The optical setting and sensitivity of our system is similar to that of traditional FACS (Figure 2B, top left and bottom left), allowing us to use the same gate settings as for FACS using FSC, BSC, and fluorescence signals excited by two lasers. We believe this approach would be effective, as the same gate setting as used for standard flow cytometry can be used. MACSQuant Tyto (Miltenyi) is a commercially available system with a disposable, fully closed cartridge. While the cartridge has a sample input chamber and collection chamber, it is not compatible with bags supplied with sample fluid and collection fluid. Our fluidic system is compatible with sterile connection of bags with sample fluid and collection fluid, which makes it easy to connect pre- and post-operation of cell isolation. Our system can implement up to three lasers, enabling us to detect two scatter signals and eight fluorescence signals. This would be effective to perform multiparametric cell isolation to collect cells of interest. However, availability of cGMP-grade reagents such as fluorochrome-conjugated antibodies is limited, and they would be expensive to have produced. After the clinical utility of multiparametric sorted cells as an infusion of cell therapy materials is shown, the reagent suppliers would enter this space with cGMP-grade reagents; then, the availability of these reagents could be improved, and the cost of these reagents could be less expensive.

In conclusion, here, we reported fully closed microfluidic chip cell sorter with a suction mechanism that can meet cGMP regulations, and its sorting performance was demonstrated. Functional analysis revealed that the sorted cells maintained their ability to proliferate, release cytokines, and maintain cytotoxicity. We believe that our system can be applied to the manufacture of cell therapies for clinical use.

## Materials and methods

### Ethics statement

Written informed consent was obtained from healthy volunteers according to the guidelines of the Declaration of Helsinki. The experimental protocol was approved by the Institutional Review Boards at Mie University School of Medicine (approval no. 3264) and Sony Group Corporation (approval nos.16-F-0009, 18-R-0015, and 19-R-0014).

### Sample preparation

PBMCs were isolated from fresh heparinized venous blood samples using density-gradient centrifugation in Ficoll-Paque PLUS (GE Healthcare) and then were stimulated using plates coated with CD3 (5 μg/mL; OKT3, Janssen Pharmaceutica, Beerse, Belgium) and RetroNectin (25 μg/mL; Takara Bio). The cells were cultured with GT-T551 (Takara Bio) supplemented with 600 IU/mL human recombinant IL-2 (Novartis), 0.2% human serum albumin (CLC Behring, Danville, CA, USA), and 0.6% autologous human plasma. The cells were used for experiments on days 7–11.

### T cell transduction

Human T cells expressing NY-ESO-1 receptor were prepared by retroviral transduction. In brief, PBMCs were stimulated using plates coated with anti-CD3 (5 μg/mL; OKT3, Janssen Pharmaceutica) and RetroNectin (25 μg/mL; Takara Bio) and were cultured with GT-T551 (Takara Bio) supplemented with 600 IU/mL human recombinant IL-2 (Novartis), 0.2% human serum albumin (CLC Behring), and 0.6% autologous human plasma. On days 4 and 5, these cells were transduced with the retroviral vector pMS3 containing NY-ESO-1 using the RetroNectin-bound virus infection method, wherein retroviral solutions were preloaded onto RetroNectin (Takara Bio)-coated plates and centrifuged at 2,000 × *g* for 2 h at 32°C, followed by expansion culture. The cells were used for experiments on day 11.

### Cell sorting

Human PBMCs were isolated using our microfluidic chip sorter. The cultured cells were washed and resuspended in sample buffer (PBS with 0.5% BSA). Cells were stained with APC-conjugated anti-CD4 antibody (RPA-T4, BioLegend) and PE-Cy7-conjugated anti-CD8 antibody (RPA-T8, BioLegend) for 60 min at 4°C, and the cells were subsequently washed and resuspended in sample buffer. FACSFlow (BD Biosciences) was used as a sheath fluid. The gate for lymphocyte+CD4+ T cells and lymphocyte+CD8+ T cells was set to include events based on FSC and BSC signals and specific fluorescence signals bound to the cells of interest.

TCR-T cells were isolated using our microfluidic chip sorter and FACSAria I (BD Biosciences). PBS with 0.5% BSA was used as a sample buffer, and FACSFlow was used as a sheath fluid. Cells were stained with APC-conjugated anti-CD4 antibody (RPA-T4, BioLegend), PE-Cy7-conjugated anti-CD8 antibody (RPA-T8, BioLegend), and PE-conjugated HLA-A∗02:01 NY-ESO-1 (157–165 aa, SLLMWITQC) Tetramer (MBL) for 60 min at 4°C. The gate for lymphocyte+CD8+tetramer+ T cells was set to include events based on FSC and BSC signals and specific fluorescence signals bound to the cells of interest. Cells were analyzed using a flow cytometer (FACSCanto, BD Biosciences). The stained and sorted cells were kept at 4°C in the dark.

### Viability assay using propidium iodide (PI)

Cells were stained with PI and then analyzed using a flow cytometer (FACSCanto, BD Biosciences). The viability was calculated as: [100 – PI+ percentage in total]. One million cells were sorted to perform this experiment.

### Cell proliferation assay

WST-1 (Roche Diagnostics) was used by following the manufacturer’s protocol. In brief, 10 μL WST-1 reagent was added to a 100 μL well, and then the plate was incubated for 240 min at 37°C. The absorbance at 450 nm (630 nm was used as a reference, and the absorbance was subtracted) was measured using a plate reader (Spectramax, Molecular Devices) every 60 min.

### Cytokine secretion assay

The human IFN-γ ELISPOT assay was performed. In brief, 96-well nitrocellulose ELISPOT plates (MAHA S4510; Millipore) were coated with anti-human IFN-γ mAb (1-D1K, ptech) overnight at 4°C, washed with 0.05% Tween 20 in PBS (PBS/Tween), and blocked with AB serum-containing culture medium for 2 h at 37°C. A total of 1 × 10^4^ TCR-T cells and 1 × 10^4^ peptide-pulsed T2 cells (NY-ESO-1 peptide-pulsed lymphocyte blast T2 cell line [ESO1-T2], MAGE-A4 peptide-pulsed T2 cell line [MAGEA4-T2]) or NY-ESO-1+ malignant melanoma cell line (NW-MEL-38) and NY-ESO-1 negative malignant melanoma cell line (SK-MEL-52) cells were placed in each well. After incubation for 18 h at 37°C, the plate was washed with PBS/Tween and then supplemented with biotinylated capture antibody (7-B6-1, Mabtech) and incubated overnight at 4°C. After washing with PBS/Tween three times, the cells were incubated with streptavidin-alkaline phosphatase conjugate in 100 μL PBS per well for 90 min at room temperature and then stained using an alkaline phosphatase conjugate substrate kit (Bio-Rad). The reaction was stopped by adding distilled water, and the spots were counted using an ELISPOT Plate Reader (ImmunoSpot, CTL-Europe GmbH).

### Cytotoxicity assay

Cytotoxicity was analyzed by the chromium release assay as previously described.^30^ In brief, 1 × 10^4^ peptide-pulsed T2 cells (NY-ESO-1 peptide-pulsed lymphocyte blast T2 cell line [ESO1-T2], MAGE-A4 peptide-pulsed T2 cell line [MAGEA4-T2]) or NY-ESO-1+ malignant melanoma cell line (NW-MEL-38) and NY-ESO-1− malignant melanoma cell line (SK-MEL-52) cells labeled with ^51^Cr were plated in triplicate cultures onto round-bottom 96-well plates in the presence of varying numbers of TCR-T cells (5 × 10^3^–1 × 10^5^ cells/0.2 mL per well) and incubated for 6 h. Control wells for determining spontaneous ^51^Cr release contained only the labeled target cells. Maximal release was determined by adding Triton X-100 (1%) to the target cells. Percentage-specific lysis was calculated according to the following formula: [(experimental ^51^Cr release – spontaneous ^51^Cr release)/(maximum ^51^Cr release – spontaneous ^51^Cr release)] × 100.

## Data and code availability

The data supporting this study are available from the corresponding author, Y.K., upon reasonable request.
